# An Overview of the Latest Metabolomics Studies on Atopic Eczema with New Directions for Study

**DOI:** 10.3390/ijms23158791

**Published:** 2022-08-08

**Authors:** Jamie Afghani, Claudia Traidl-Hoffmann, Philippe Schmitt-Kopplin, Matthias Reiger, Constanze Mueller

**Affiliations:** 1 Department of Environmental Medicine, Medical Faculty, University Augsburg, 86156 Augsburg, Germany; 2 Chair of Analytical Food Chemistry, School of Life Sciences, Technical University of Munich, 85354 Freising, Germany; 3 ZIEL-Institute for Food and Health, Technical University of Munich, 85354 Freising, Germany; 4 Christine Kühne Center for Allergy Research and Education (CK-CARE), 7265 Davos, Switzerland; 5 Institute of Environmental Medicine, German Research Center for Environmental Health, Helmholtz Zentrum München, 85764 Neuherberg, Germany; 6 Research Unit Analytical BioGeoChemistry, German Research Center for Environmental Health, Helmholtz Zentrum München, 85764 Neuherberg, Germany

**Keywords:** atopic eczema, atopic dermatitis, dermatitis, metabolomics, skin, lipidomics

## Abstract

Atopic eczema (AE) is an inflammatory skin disorder affecting approximately 20% of children worldwide and early onset can lead to asthma and allergies. Currently, the mechanisms of the disease are not fully understood. Metabolomics, the analysis of small molecules in the skin produced by the host and microbes, opens a window to observe the mechanisms of the disease which then may lead to new drug targets for AE treatment. Here, we review the latest advances in AE metabolomics, highlighting both the lipid and non-lipid molecules, along with reviewing the metabolites currently known to reside in the skin.

## 1. Introduction

Atopic eczema (AE), also known as atopic dermatitis, is an inflammatory skin disorder that affects approximately 20% of children worldwide and is increasing in prevalence [1,2,3,4,5,6]. AE can naturally resolve prior to adulthood, but for approximately 2–4% of individuals, it is a life-long condition [7]. Additionally, AE onset is also not limited to children, and 26.1% of AE-afflicted adults developed the disease in adulthood [8]. Even though AE can go into remission past childhood, it can also begin a cascade of immune reactions later on in life (such as asthma and allergies) in a process termed the “atopic march” [9]. AE is also associated with an increased incidence of heart disease, and heart failure is 70% more common in people with severe AE [10]. One prevailing AE symptom is skin itchiness [11], which leads to scratching and, in most cases, mechanical skin barrier damage and the exacerbation of inflammation. This starts a vicious cycle of inflammation, further itching, further scratching, and continuous damage to the skin. This AE state also extends deeper than direct physical suffering, as patients have a significantly reduced quality of sleep and, consequently, often additionally suffer from depression and anxiety, leading to an overall reduced quality of life [11].

**Figure 1:**
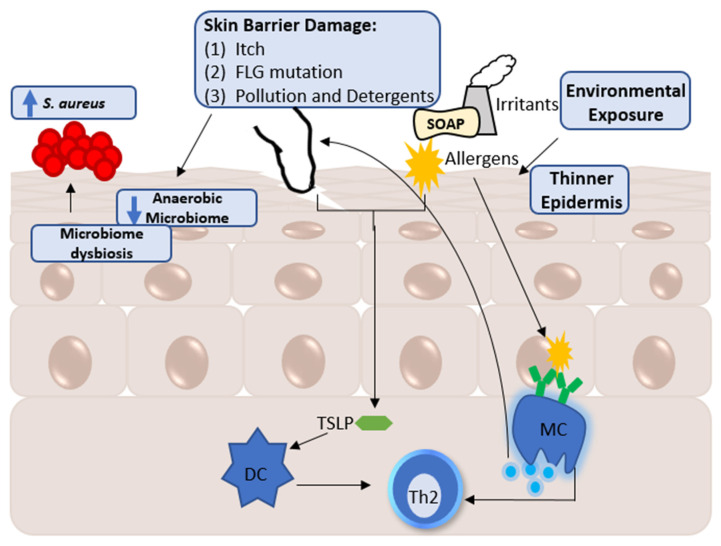
Model of AE development. The environment and the host could both drive the development of atopic eczema (AE). AE is characterized as having a disrupted skin barrier which could be induced through itching, filaggrin (FLG) mutation, and environmental exposures. The disruption then allows for oxygen to penetrate the skin, resulting in a decrease in the anaerobic microbiome and providing a new niche for bacterial growth. Microbial dysbiosis within AE could be a result of this niche, or could be the result of changing pH, salt levels, and/or other factors within the skin. This dysbiosis then provides the opportunity for *S. aureus* to overgrow. In addition, both scratching and allergens result in keratinocytes releasing cytokines, such as the thymic stromal lymphopoietin (TSLP). The cytokines then lead to the induction of a T-helper cell 2 (Th2) response through dendritic cell (DC) recruitment. AE non-lesional skin also has a thinner epidermis, allowing for easier penetration of allergens and irritants. These allergens can then activate mast cells (MC), resulting in the secretion of tryptase and histamine and thereby inducing itching.

AE has been the subject of many reviews, and a more in-depth review of the currently known mechanisms of AE can be provided by [12] with an update on the pathogenesis and therapeutics found at [13]. Here, we will briefly overview what is known about AE and highlight its genetic predispositions, environmental factors, and immunological observations (summarized in Figure 1).

Despite the large breadth of the literature describing AE, the mechanisms are still unknown [12] and often disputed, especially in the case of whether the host or the environment is the initial or main driving stimulus of AE. People can be predisposed to AE by both their genetics and their environment [5,14]. Some of the genetic predisposition of AE is due to the mutation of skin barrier proteins such as filaggrin, loricrin, and involucrin [15,16,17,18], with filaggrin (FLG) mutation being more common relative to loricrin and involucrin [19]. Although a genetic predisposition within barrier proteins would explain the development of the disease, this predisposition is not universal. In the case of FLG, genetic predisposition does not entirely account for the majority of afflicted Europeans [12], suggesting that other proteins or mechanisms may be involved.

While this lack of agreement for FLG does not discount another undiscovered host-related factor as the main contributor to AE development, it does lend credence to the idea of environmental stimuli being the universal driving factor. Heavy use of soap and exposure to pollution can both lead to AE development as both of them damage the microbial skin barrier [20,21,22,23,24]. This is indicated by a decrease in antimicrobial peptide secretion in AE patients [25] and the reduction in their anaerobic microbiome [26]. Damage to the microbial barrier also provides a new niche for pathogenic bacteria overgrowth. One such pathogen is *Staphylococcus aureus*, which has been shown to be overgrown in patients with AE [27] and is a predictive factor for severity [28]. This hypothesis of AE being a microbial dysbiosis disease is enticing, but does not explain why some individuals who are exposed to the same stimulants do not develop AE. It is very likely that a compounded stimulus drives AE, where these two factors, both host and environment, are dependent on each other.

As AE is described as an inflammatory disease, naturally, the immune system also plays a role in the mechanism of AE and provides a connection between the host and the environment. Physiologically, AE-uninvolved, non-lesional skin has a thinner epidermis in comparison to healthy skin and those with other types of dermatitis [29], which allows easier exposure to the skin’s immune system. Stimulation, whether through allergens or irritants, makes keratinocytes release cytokines such as thymic stromal lymphopoietin (TSLP) to recruit dendritic cells, leading to the T helper cell 2 (Th2) response [30]. AE is characterized as a Th2-cell-dominated immune response [31,32]. Acute and chronic AE can be further stratified by their Th cells, where Th2, 17 and 22 are associated with acute AE and Th 1, 2, and 22 with chronic AE [33]. In addition, as hinted at by the process of atopic march, the allergy-related cytokine IgE is upregulated in the patients of AE [34,35]. IgE can activate mast cells, and mast cells are more abundant in AE lesions, but this accumulation can also occur independently of IgE presence [35]. While it can be debated whether the root cause of the disease is host-dependent or purely environmental, recent advances in the field shows it is likely a combination of factors—implicating both the host and the environment in the onset of AE.

Metabolomics can provide unique insight into the mechanisms of this disease by defining the low molecular weight compounds involved in the AE biological system. In essence, the metabolome provides a promising window into the mechanics of the body’s microbe–host interaction [36]. Recently, skin metabolomics has become the subject of increased interest in the scientific community due to its ability to describe not only skin disease but also diseases such as Parkinson’s disease, lung cancer, and cystic fibrosis [37]. In this review, we cover several articles highlighting the recent advancements in the metabolomics of skin disease, with a focus on AE. More specifically, this review covers the factors influencing the skin’s metabolism and the current state of the metabolic understanding of AE.

## 2. Factors Influencing the Skin Metabolome

The skin is the body’s largest organ and has a tremendous impact on both our physical and mental health [38,39]. In the skin, metabolites are produced through (1) the excretions from various glands; (2) the breakdown, secretion, or modification of the skin’s constituent cells including resident microbiota; or (3) exposure to the environment. The metabolites within the skin are not limited only to those produced within or by the skin itself, but other organs such as the immune system and microbiome also play a role. For more general overviews of the skin’s immune system and the microbiome’s role within the skin, see [40,41,42,43,44,45].

The skin as a whole serves as our barrier to the outside environment and, because of its high degree of interaction with the environment, several external factors must be taken into account for direct-skin metabolomics. The skin’s metabolome can be affected directly through the skin barrier, like in the case of cosmetics [46], pollution [47], and UV light [48], but also through gut-to-skin interaction, e.g., in the case of caffeine consumption [49]. In addition, intrinsic host factors play a role in the skin metabolome. One such factor is age. Along with the loss of firm collagen filaments and coenzyme Q10 in older skin, other metabolites previously thought to be unrelated can be affected [50]. Another potentially confounding host factor is biological sex, but this confoundment appears to depend on the skin matrix used, where different sexes have distinct metabolomes in sweat [51] but not in surface-stratum corneum sampling [38]. These environmental and host influences on the skin metabolome can sometimes benefit the medical community by providing explanations for disease development, treatment effectiveness and side effects. Small molecules such as glucose can be used as potential biomarkers for disease or elucidate deeper mechanisms present in skin disease [52,53]. Additionally, the skin metabolome does not change only in skin diseases but can even reflect changes from Parkinson’s disease [54,55], cancer [56], and many other disorders, as reviewed in [37]. There have also been cases where skin metabolomics was used for drug monitoring [57,58]. All of the environmental and innate effects of skin immunity on metabolism display the high interconnectivity of the skin, and the potential use of the skin as a highway for non-invasive medical screening.

### A Note Regarding Skin Metabolomic Collection and Processing

As skin metabolomics is beginning to grow as a field, we have the unique opportunity to improve the standardization of studies for future research. Although studies may overlap in chemical targets, many confounding factors can play a role in the results. Without accounting for them, poor cross-comparisons across both studies and samples will be seen. For example, in urine metabolomics the levels of hydration and other host factors can play a role in the concentrations of metabolites [59,60]. To account for this, urinary metabolomics uses creatinine and a host of other normalization factors and methods [60], and the skin matrix should not be excluded from such standardization. Additionally, by looking at the results of other matrixes, there are a host of potential factors such as temperature and sample processing time [61] for lipids, along with time-of-day for amino acids and phospholipids [62], that can influence sample- and study-level differences. For skin metabolomics, one such factor was already discovered, where the sampling method for the skin can influence the composition of the captured metabolome [38]. Currently, we have the unique opportunity to determine the causes of variability across studies before a “boom” in the field of skin metabolomics. Potential sources of variability could be because of differences (1) that may exist across the skin’s layers, (2) within the various pockets of the skin, such as sweat glands and interstitial fluid, and (3) in the sampling location. There are various research groups that have begun to explore [38,63] the complexities and influences within skin metabolomics, not only at the host level, but also at the environmental level, since the epidermis is primarily our first barrier protecting us from the outside environment. Because of these open questions, these are exciting times for skin metabolomics, which will lead to a bright future in further understanding our largest organ, especially in disease development.

## 3. Current Understanding in the Metabolomics of Atopic Eczema

Constructing a universal picture of AE for all affected individuals is difficult due to the variety of endotypes involved in AE [34]. As hinted at by the process of atopic march, allergy-related cytokine IgE is upregulated in patients with AE [34,35]. Many have supposed that the atopic state is due to the lack of childhood stimuli to create an IgE-tolerant or allergen-tolerant phenotype because of the disease’s origin early in the patient’s life [64]. This was first termed the hygiene hypothesis [65], later renamed the “microbial exposure hypothesis” [66]. It has since been confirmed that early-life stimulation in less polluted yet “dirtier” rural environments decreases the incidence of AE [67]. The IgE phenotype is not universal across all individuals and is suggested to be one endotype of AE. Immunologically speaking, there are two endotypes: IgE-mediated (extrinsic) or non-mediated (intrinsic) [12,34], and biological race. Within the biological race endotype, the differences include a higher Th17 response in Asian AE sufferers that is completely absent in African AE and only slightly present in European AE [34]. In addition to immunologically characterized endotypes, there are also FLG-deficient or -present endotypes and *S. aureus*-influenced or -independent [34] endotypes. Because of the various endotypes of AE, there is a need for further metabolic profiling, because small molecules are often the signals between immune, genetic, and bacterial systems. Many scientists have attempted to illuminate the mechanisms of AE to allow for the more precise diagnosis, understanding, and treatment of patients through metabolomic profiling. Here, we review the latest advances in the AE metabolomic world.

### 3.1. Findings within the Skin

A large portion of AE metabolomics research is focused on lipids, reviewed from the epidermal lipid perspective in Bhattacharya et al., 2019 [68] and with a greater focus on the cornified layer in van Smeden and Bouwstra 2016 [69]. The focus of this review is to not only cover lipids, but also the metabolome as a whole. Seeing that AE is described as a skin disease, it is highly relevant to determine the metabolite presence directly at the location of the disease. These studies—focused on the direct skin monitoring of metabolites, summarized in Table 1—show a clear distinction between the small molecules present in AE as compared to healthy skin [70]. Among those changed, on the lipidomic side, there are acylcarnitines and glycerophospholipids, and on the non-lipid side, there are amino acids and their derivatives [70]. Because of the typical locations of these metabolites within the skin, it suggests that the skin as a whole is modified in AE, instead of in one particular subcompartment. Metabolomics profiles can change according to the sampling location for healthy skin [63]. This is also the case for ceramides in non-lesional AE skin, where the forehead, the cubital fossa, and the proximal lower forearm have unique profiles [71]. Although the location of skin sampling can influence the metabolome, AE is a disease that affects the entire body, so metabolites that truly reflect the disease should be consistent regardless of location.

The current knowledge of AE endotypes in relation to metabolomic profiles is still developing, with six papers published so far, each focused on an individual endotype, and three of the six focused on the skin. Starting with the filaggrin endotype, there is no initial correlation between the lipidome and filaggrin (FLG) mutation [71], and the FLG genotype does not alter the stratum corneum lipid structure [74]. Granted, the lipidome studied in [71] did not cover the correlation of long-chain ceramides with FLG but instead only the short-chain ceramides, free fatty acids, and cholesterols. This potential lack of association should be confirmed in an untargeted lipidomics study. Heterozygous FLG (FLG^+/−^) mutation in AE does result in an increase in arachidonic acid due to the increased breakdown of linoleic acid and breakdown of phospholipids from IL-1β-induced PLA_2_ activity [74]. The increase in arachidonic acid is not seen in wild-type (FLG^+/+^) AE individuals; therefore, FLG might play a role in controlling arachidonic acid levels. Because arachidonic acid promotes inflammation, FLG (FLG^+/−^) mutation may play a role in the inflammation of non-lesional AE skin through the production of arachidonic acid [74]. This adds a potential mechanism that may explain the FLG endotype, where FLG or its products are inhibitors of arachidonic acid production. Aside from the lipids, and in spite of FLG being metabolized to form amino acids, currently there are no skin studies stratifying the amino acid changes in AE [70] to the FLG endotype.

**Figure 2:**
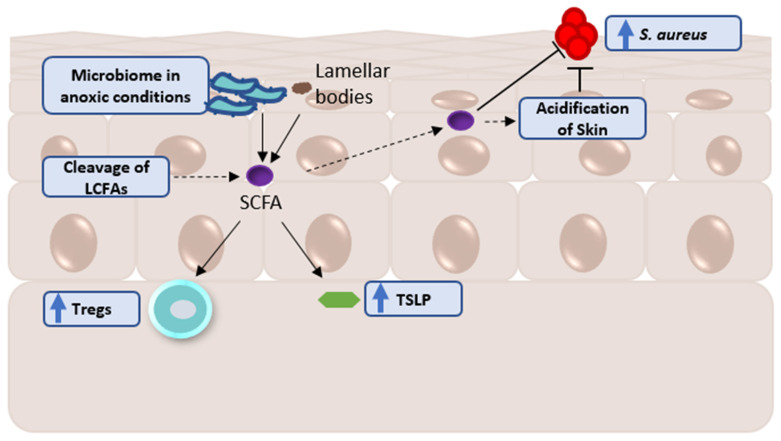
Diagram of shorter-chain free fatty acids (SCFA) within atopic eczema. Free fatty acids are produced from the breakdown of the lamellar bodies [81,82], and the lysis of the skin’s proteins and sebum by *Cutibacterium acnes* [83,84], and a select few are produced by the microbiome under anoxic conditions by the fermentation of carbohydrates [85,86,87,88,89]. These free fatty acids contribute to the acidification of the skin, which in healthy individuals is around 4.7 [90]. This acidification discourages *S. aureus* growth, and the production of SCFAs inhibit the other growth of pathogenic sub-strains [79]. Resident skin bacteria can also use the SCFAs to interact with the host’s immune system through inducing T regulator cells (Tregs)—by decreasing the transcription of *il6*—and TSLP [91,92,93,94]. Studies in other organs suggest that SCFAs can inhibit dendritic cells’ expression of lipopolysaccharide-induced cytokines (IL-6 and IL-12p40) [95] and activate macrophages, neutrophils, and monocytes through the G-protein-coupled receptor FFAR2 [96]. Although well studied in other organ systems, the SCFAs’ mode of action in the skin is still unclear. Symbol: ↑ (increase) and a dotted arrow (theoretical mechanism).

AE-afflicted individuals with the endotype of *S. aureus* colonization have a different lipidome as compared to *S. aureus*-absent individuals [75]. More specifically, AE skin has an increase in shorter-chain free fatty acids (SCFAs) [71], but this effect appears to be *S. aureus*-dependent. Saturated SCFAs are negatively correlated with Staphylococci [71], and their long-chain counterparts are decreased in *S. aureus*-colonized AE skin [75]. The connection with SCFA is further suggested through the inverse correlation of n-6 FA with disease severity [76], because it is known that *S. aureus* abundance can be a predictor of increasing AE severity [28]. The inverse correlation between *S. aureus* and SCFA may be due to the inherent effect that shorter fatty acids can more easily traverse the skin to acidify it, and *S. aureus* does not grow well in acidic healthy skin pH conditions. This means that a decrease in the level of long-chain free fatty acids (LCFA) may be due to cleavage to create shorter chains that allow for better mobility within the skin, and the cleavage is upregulated in AE skin to discourage *S. aureus* growth. This may also be due to direct inhibition, since SCFAs have been shown to inhibit the overgrowth of pathogenic S. aureus sub-strains [79]. The presence of ceramides also suggests inhibitory effects of *S. aureus* overgrowth in AE, where long-chain ceramides are downregulated in *S. aureus*-infected AE skin [75]. Although total ceramide levels are often reported as decreased in AE patients, this appears to be chain-length-dependent, and short-chain ceramide levels have been shown to be increased in AE patients [71,75], which may be indicative of future or current *S. aureus* presence, but further research is needed for confirmation. The relationship between ceramides and *S. aureus* may be an off-shoot of the original association between FFA and *S. aureus*. Since LCFA levels are decreased in *S. aureus*-colonized AE skin, they are no longer available for the de novo synthesis of ceramides by combination with sphinganine. This relationship between decreasing levels of long-chain ceramides [75] and AE could be modulated through dupilumab, a drug targeting IL-4 receptor alpha chain and known to decrease levels of *S. aureus* [80]. Dupilumab has been shown to alleviate the severity of AE and was shown to result in an increase in the level of C26 ceramide [78]. Unfortunately, this increase appears to be transient and more a factor of increasing stratum corneum hydration [78]. However, it does raise the question of the mechanism between dupilumab treatment and *S. aureus* reduction: could it be possible that C26 ceramide provides an inhibitory effect on *S. aureus*? Dupilumab could be simply reducing *S. aureus* abundance through skin hydration, but further non-targeted studies on dupilumab and *S. aureus* would be beneficial. Overall, these observational studies shine a bright light on our further understanding of the mechanisms of *S. aureus* overgrowth in AE (Figure 2) and the endotypes involved in AE (summarized in Table 2).

As of now, a large portion of direct skin AE studies focus on the changing ratio of the lipid components of the skin, due to its predominance in the structural integrity of the skin’s barrier. Lipids are not the only metabolite present in the skin, and more studies are beginning to go beyond lipids to other molecules in order to explain the effects seen in AE. One such effect is the negative impact sweat has on AE. Contrary to skin measurements [99], sodium and salts were found to be similar between HE and AE individuals’ sweat [53], and the presence of glucose within sweat positively correlated with AE severity [53]. This increase in glucose appeared to be specific to acute inflammation in comparison to chronic AE inflamed and non-inflamed skin [53], suggesting a role in early AE development. The increase in glucose did not correlate with an increase in the downstream fermentation product lactate, suggesting that it was not a cessation of the TCA cycle [53]. It is possible that this lactate does not come from human cells but is a product of microbial fermentation. This explanation would also support the decrease in pyruvate seen, where the TCA cycle may be downregulated, resulting in less pyruvate [53]. If true, this increase in glucose would signify a loss of SCFA, which can be used to prevent inflammation through TREGs [91]. Nevertheless, in AE skin there is an upregulation of the glucose transporter GLUT2 [53], and further research needs to be conducted to determine if the regulation of glucose levels can have positive effects on AE treatment and its relationship with SCFA. In addition to a spike in relative glucose levels, there is also an increase in amino acids in AE lesional skin [70]. Because amino acids can be produced through the breakdown of filaggrin, it would be interesting to see if this spike in amino acids is dependent on the FLG mutant endotype of AE. Lastly, although sodium levels were found to be similar within HE and AE sweat, this finding does not discount the potential for variation within other subcompartments within the skin, but instead encourages the comparison with other sampling methods to confirm such results.

### 3.2. Findings within the Blood

The difference in the metabolomic ecosystem between AE-afflicted and healthy individuals seen within the skin was also found to be true for the circulatory system [97,100,101], with a summary shown in Table 3. The correlation between the skin and blood matrix is corroborated by the study of Agrawal et al. [102], where sweat metabolites and serum have been found to overlap in lipid profiles. However, this is not universal for all metabolites. A study by Töröcsik et al. [72] found that lipid arachidonic acid was significantly changed in skin biopsies but not in serum, and further non-targeted research should be performed to determine the overall metabolic overlap between these two matrices [37].

According to unsupervised statistics, the untargeted AE and HE metabolome was not separated within the blood [100], but instead differences appeared to be more on the singular molecule-to-molecule basis. It is also possible that global differences were better parsed out after stratifying for the individual endotypes within AE. Deficiency in cytoskeletal protein DOCK8 can result in AE, but the metabolic profiles are distinct between DOCK8-deficient and AE WT individuals [101]. This implies the potential for metabolomics to differentiate between genetic subgroups of AE, such as with the FLG endotype. The filaggrin mutation has been shown to display a unique plasma metabolome and filaggrin-associated metabolites correlate with high IgE levels [97]. This suggests a filaggrin-IgE relationship, which was also seen in the case of omalizumab treatment, and although it is a known drug for urticaria, omalizumab is currently under consideration for treatment in AE. In this study, treatment against IgE is only effective in filaggrin wild-type individuals [98]. It also suggests that these endotypes might not be separated and instead combine into one greater endotype, filaggrin-IgE. Amino acids are increased in AE skin [70], and assuming the correlative relationship across the different matrices, treatment against IgE (omalizumab) in filaggrin wild-type individuals can decrease serum amino acid levels [98]. This suggests that amino acid production may be regulated through the adaptive immune response. Omalizumab can also decrease levels of acylcarnitines [98], but this may have a negative effect on the metabolism of AE patients because total acylcarnitines are typically decreased in AE patients [100]. A double-blind study ought to be performed to determine if omalizumab is the cause of acylcarnitine decrease or if this is an inherent effect of AE progression over time.

In regard to the *S. aureus* endotype, it has been suggested that bacterial secretions—specifically SCFAs—play a protective role against AE development, but the plasma of AE individuals displayed increased succinic acid and lactic acid levels [97]. This suggests that either the bacteria are trying to compensate for *S. aureus* overgrowth by increasing the production of SCFAs, thereby lowering the skin pH, or that SCFAs do not play a regulatory role against AE development. The dynamics between SCFAs within the blood and skin should also be studied to determine if this difference is inherent in the difference between the matrixes used. To the authors’ knowledge, no studies have directly addressed the *S. aureus* endotype in relation to the AE blood metabolome or lipidome. In addition to studies on the diversity between individual adult AE endotypes, there have also been many studies on the metabolite expression in infants.

### 3.3. Infantile AE Findings as a Precursor for the AE Metabolome

Affected infants have a unique immunological profile compared to adults with AE [34] and this may also be the case for the infantile metabolome (Table 4). In the case of lipids, SCFA appear to be a conserved metabolite group seen in both infants and adults. For infants, SCFA, butyrate and valerate are present in persistent AE and HE cases, but their levels are lower in individuals with transient AE who later recovered [104]. SCFAs are suggested to protect AE through Tregs or the modulation of *S. aureus* growth (Figure 2). These results suggest a protective effect of these SCFA in short-term AE cases but a dysregulation or inability to compensate and protect the skin from inflammation in chronic AE. Lower levels of SCFA butyrate and propionate in early life (~6 mo. after birth) are associated with AE development within 2 years of age [105], further supporting the protective effect of SCFA against the development of AE. This association with SCFA is possibly a result of early weaning where SCFA-producing bacteria from breast milk have not yet been fully established within the gut [105]. Another possible cause of early-stage AE is high amounts of long-chain saturated fatty acids within the breast milk of mothers with AE children [106]. Exposure to long-chain saturated fatty acids in milk can result in an accumulation of type 3 innate lymphoid cells (ILC3) within the gut [106]. These lymphoid cells can then migrate to the skin, thereby triggering the first inflammation of the AE cycle [106]. SCFAs are not the only conserved metabolite type seen in AE individuals across the ages. Hexoses, including glucose, are present in high amounts in both newborns and infants [107]. Glucose drives inflammatory expression in early-stage AE [107] and is associated with the expression of bacterial virulence factors within the gut of infants with AE [105]. Amino acid expression is also suggested to have a protective effect against infantile AE with an inverse correlation with inflammasome expression [107]. Overall, this highlights several conserved mechanisms that occur across both early-stage and advanced stage AE. 

Besides conserved mechanisms, studying AE at the infantile level allows for a unique opportunity to observe the abiotic environmental impacts on AE development. Pollution, such as parabens, has been suggested to be a major factor in the development of AE. Lee et al. 2021 [108] followed a cohort of 455 children to determine the association between propyl-parabens, aeroallergen sensitization, and AE. Propyl-parabens were associated with AE severity, but not with the disease as a whole, suggesting that other pollutant factors may be the cause of AE development or that pollution is not actually a cause but rather an aggravating factor in AE [108].

In regard to the endotypes of AE (listed in Table 2), there is currently only one study conducted with infants focused on the IgE endotype. Infantile AE can be subtyped into intrinsic and extrinsic profiles, where low and high IgE have unique metabolic signatures. It has been shown that the presence of SCFAs, such as lactic acid, correlates with IgE levels [109]. Infants with allergies and AE have also been shown to have a distinct metabolic endotype from infants with AE but without allergies. These changes can be arranged along a spectrum of similarity from healthy infants, to AE-afflicted infants without allergies, to AE-afflicted infants with allergies [105]. These mechanisms suggest (1) the conservation of the processes between infant and adult AE, (2) that the initial driving factors are best seen in further characterization studies on transient and persistent AE at the beginning of life, and (3) the possibility that metabolomics may eventually be able to detect the initial stages of the progression of the atopic march in individuals in which AE has appeared to recede.

**Table 4:** Summary of AE metabolomics studies with infant samples. Abbreviations: AE (atopic eczema participants), LS (lesional skin), NL (non-lesional skin), HE (healthy participants), P (psoriasis participants), SCSFAs (short-chain saturated fatty acids), LCSFAs (long-chain saturated fatty acids), FA (fatty acids), ADMA (asymmetric dimethyl-arginine), C (chain length). Co. (cooperation), FLG (filaggrin), FFA (free fatty acids), UT (untargeted), IV (ichthyosis vulgaris), PBMCs (peripheral blood mononuclear cell), PUFA (poly-unsaturated fatty acids), and PCA (principal component analysis). Symbols: ↑ (increase) and ↓ (decrease).

References	Matrices	Target	Sample Size	Findings
[109]	Serum	Eicosanoids and UT	41 AE 22 HE 42 AE 23 HE	Metabolomics profiles separated according to IgE levels ↓ Glycine in AE vs. HE ↓ Taurine in AE vs. HE ↑ Unsaturated fatty acid in AE vs. HE ↑ Carnitines, FFA, sphingomyelins, and lactic acid in high IgE AE vs. low IgE AE and HE
[110]	Urine	UT	20 AE 12 HE	By supervised statistics, there is a prominent distinction between AE and HE ↑ Creatinine, creatine, citrate, formate, 2-hydroxybutyrate, dimethylglycine, and lactate in AE vs. HE ↓ Betaine, glycine, and alanine in AE vs. HE
[111]	Tape strips	Lipids	28 AE 32 HE	↓ Glyceroglycolipids in AE LS vs. HE ↓ Sphingomyelin in AE LS vs. HE ↑ Glycerophospholipids in AE LS vs. HE
[104]	Fecal matter	SCFA	24 AE 33 HE	↑ Butyrate in HE and persistent AE vs. transient AE ↑ Valerate in HE and persistent AE vs. transient AE No difference in acetate and propionate between the groups
[108]	Urine	UT	455 Children	Propyl-parabens presence is associated with aeroallergen sensitization but not with AE Propyl-paraben is associated with AE severity ↑ Amino acids in general within the high propyl-paraben group ↑ Picolinic acid in high propyl-paraben group ↓ 2-palmitoylglycerol in high propyl-paraben group
[107]	Serum	Metabolites in Biocrates Absolute IDQ^®^ P180 kit	495 Newborns 449 1-year-olds	↑ Hexose levels in newborns and 1-year old AE Amino acids are negatively correlated with inflammasome expression Lysophosphatidylcholines negatively correlate with inflammasome expression
[106]	Breast milk of AE mothers	UT	75 AE 75 HE	↑ LCSFA in AE mothers’ vs. HE mothers’ milk
[105]	Fecal matter	SCFA and UT	33 AE 30 HE	Allergy sensitization is an endotype of AE ↓ Butyrate and propionate in infants that later developed eczema
[112]	Plasma	Vitamin D	4327 2-year-olds	Vitamin D does not predict the development of AE

## 4. Future Directions

Currently, the field of metabolomics and skin is like a barren but fertile land, where there is great potential for profitable discoveries and the establishment of new disciplines, along with the risk of confounding factors and a lack of standardization in measurement and sampling techniques. Should these problems not be rectified, the great potential of the skin metabolome will be lost through the lack of unifiable data across individual researchers. However, if the discipline can unify itself and avoid these stunting effects, it has the potential to grow into an indispensable part of the holistic understanding of human biology, as well as a massive source of medical innovation.

One such innovation could target atopic eczema (AE), and this review covers the recent advances in the metabolomic profiling of AE for the skin, blood and infantile metabolomes. Despite the moderate breadth of the literature on the subject, much more needs to be explored. The majority of the research is primarily focused on the lipidomic profile, and further studies extending beyond lipids are recommended in order to understand the full breadth of metabolism within AE-afflicted individuals. One example is the study of volatile organic compounds (VOC). AE has been suggested to have a unique scent, i.e., VOC profile, and VOCs could be the best method to create an official laboratory diagnostic for AE.

In addition, many of the studies have covered a wide range of matrices, and further cross-organ studies would be recommended to highlight the full translation of direct skin metabolomics to the other biological systems, and to elucidate further factors involved in the progression of AE. Along with cross-organ studies, the metabolomic shifts that occur within the different skin departments should be explored. This would aid not only in determining comparability across different skin matrices, but also in understanding compartmental influences on disease. Of note is the subcutaneous tissue because of its varying thickness across body sites, which may explain metabolomic locational differences, but also because it has a direct connection to the circulatory system and immune system [113]. 

The subcutaneous tissue may also play a predominant role in AE through its connection with salt. The skin pH of AE is less acidic, and surprisingly, the pH difference is not due to filaggrin deficiency [86,114]. The pH rise in AE skin could be due to the change in sodium content within the skin. Moderate to severe AE is associated with hypertension [115], and the skin is a reservoir of sodium with its levels being positively associated with blood pressure, as reviewed in [116]. Sodium levels within the skin are correlated with dietary salt levels [117] and skin pH is correlated with AE severity, as measured through SCORAD [118]. If sodium is the main contributing factor to the AE skin pH change, then pH-induced severity in AE could be modulated through dietary salt levels. The salt–AE connection is further supported by the significantly higher levels of salt found in AE lesional skin [99]. In addition to pH modulation, salt could also play a role in the AE immune response through the induction of the T helper cell 2 response [99]. To our knowledge, this is the first suggested mechanism connecting diet, blood pressure, and AE severity. 

Many of the studies reviewed here covered less than 25 participants. Through further collaboration between these research groups, as well as merging datasets and participant pools, we might begin to see large-scale metabolomics research enterprises that reach the necessary statistical power for a final diagnostic biomarker of AE. Although it was found that there are body-site-based differences in a targeted lipid study of AE-afflicted individuals [71], this research needs to be broadened to determine if location is relevant to lesional metabolism and the complete skin metabolome. This research will determine if body site is a confounding factor that needs to be accounted for when developing a metabolite-based diagnostic of AE skin. It will also help determine if the mechanism of AE is unique to body location. Because non-lesional samples can be taken in close proximity to the lesion, metabolomic imaging should be performed to determine the true line of where the lesion ends and non-lesional skin begins. In addition to determining the confounding effects of location, further longitudinal research is necessary to understand the mechanisms behind the atopic march. Recent research highlighted the importance of mast cells in the regulation of atopic itching and development [35]. In combination with the knowledge that arachidonic acid mediators and histamine levels are increased in AE [119,120], and that its mediators can influence mast cells to release histamine [121], further research should be conducted to determine if this is another pathway involved in AE inflammation. Overall, this is an exciting time to be performing metabolomics research in the field of eczema, and this research will be instrumental in further understanding AE and providing novel treatments.

## Figures and Tables

**Table 1:** Summary of AE metabolomics studies with skin samples. Abbreviations: AE (atopic eczema participants), LS (lesional skin), NL (non-lesional skin), HE (healthy participants), P (psoriasis participants), SCSFAs (short-chain saturated fatty acids), LCSFAs (long-chain saturated fatty acids), FA (fatty acids), ADMA (asymmetric dimethyl-arginine), C (chain length). Co. (cooperation), FLG (filaggrin), FFA (free fatty acids), UT (untargeted), and IV (ichthyosis vulgaris). Symbols: ↑ (increase) and ↓ (decrease).

References	Matrices	Target	Sample Size	Findings
[70]	Skin biopsy	Metabolites in Biocrates Absolute IDQ^®^ P180 kit	15 AE 17 HE	↑ Putrescine in AE LS as compared to AE NL and HE ↑ ADMA in AE LS as compared to AE NL ↑ Amino acids in AE LS as compared to AE NL and HE ↑ Sphingolipids in AE LS as compared to AE NL and HE
[71]	Tape strips	Metabolites in Waters Co. TrueMass^®^ Stratum corneum metabolon lipid panel	10 AE 10 HE	FLG is not associated with changing lipid composition of AE ↑ Short-chain ceramides in AE ↑ FFA in AE ↑ Cholesterol-sulphate in AE SCSFAs negatively correlated with *Staphylococcus* presence Several ceramide species positively correlate with *Staphylococcus* presence
[72]	Skin biopsy	FFA, eicosanoids, and docosanoids	3 AE 6 HE	↑ Arachidonic acid in AE LS and NL vs. HE ↑ PUFA-hydroxy metabolites in AE vs. HE ↑ 5-lipoxygenase-derived metabolites in AE LS ↑ in cyclooxygenases metabolites in AE vs. HE
[53]	Sweat	UT	21 AE 6 Other dermatoses 10 HE	Glucose positively correlates with AE severity ↑ Glucose in AE with acute inflammation versus chronic inflammation and HE No difference in lactate between AE and HE No difference in sodium and salt content between AE and HE
[73]	Sweat	Lipid mediators	11 AE 12 HE	↑ C30-40 NS ceramides in AE ↑ C30-40 NS ceramides in AE ↑ C18:1 sphingosine in AE ↑ 10-nitrooleate in AE
[74]	Skin Biopsy	FFA, Eicosanoids, and Docosanoids	18 AE 3 IV 14 HE	Stratum corneum lipid structure alteration is not related to the FLG genotype ↑ Arachidonic acid in AE FLG(+/−) compared to AE FLG(+/+), HE, and IV ↑ Hydroxy fatty acid HETE-12 in AE FLG(+/−) compared to other groups
[75]	Stratum corneum lipid extraction	Lipids, ceramides, cholesterols, FFA, and triglycerides	27 AE 15 HE	↓ FFA in AE with *S. aureus* growth ↓ Triglycerides in AE with *S. aureus* growth Cholesterol is not associated with *S. aureus* growth ↑ Short-chain ceramides in AE versus HE ↓ Long-chain ceramides in AE with *S. aureus* growth vs. AE without *S. aureus* Certain ceramides correlate with *S. aureus* presence
[76]	Skin biopsy	Lipids	15 AE 9 HE	↑ Arachidonic acid AE LS compared to AE NL ↑ SCSFAs in AE ↓ LCSFAs in AE n-6 FA inversely correlated with disease severity in AE NL ↑ Phospholipids in AE (NL and LS) compared to HE
[77]	Interstitial fluid	Arachidonic-acid-derived mediators	16 AE 9 P 12 HE	↑ LBT4 within AE LS compared to NL and HE, with similar results in P LBT4 has no correlation with AE disease severity No significant difference in PGE2 levels between groups
[78]	Tape strips	Ceramides	10 AE 10 HE	↑ C26 ceramide within non-lesional and lesional skin treated with dupilumab C26 ceramide did not correlate with reduction in AE severity in dupilumab-treated AE participants C26 ceramide did correlate with stratum corneum hydration

**Table 2:** Overview of adult metabolite profiles according to established AE endotypes. Abbreviations: u.s (unstudied). The upward arrow (↑) indicates an increase in the metabolite relative to the first category and the downward arrow indicates a decrease (↓) in the metabolite relative to the first category.

Endotype	Categories	Matrix	Metabolite Profile Relative to First Category
IgE	Mediated (extrinsic) vs. non-mediated (intrinsic)	Blood	↑ Isopropanol [97] ↑ Threonine [97] ↑ Betanine [97] ↑ Creatinine [97] ↑ Dimethylamine [97]
FLG	Deficient vs. non-deficient	Skin	No broad differences in lipid composition [71,74] ↓ Arachidonic acid [74] ↓ Hydroxy fatty acid HETE-12 [74]
		Blood	↓ Glycerophospholipids [98] ↓ Sphingomyelin [98] ↓ Amino Acids [98] ↓ Acylcarnitines [98] ↑ Isopropanol [97] ↑ Iso-butyrate [97] ↑ Isoleucine [97] ↑ Tyramine [97] ↑ Histidine [97] ↑ Threonine [97]
Staphylococci	Present vs. absent	Skin	↓ SCSFAs [71] ↑ Ceramide subspecies AS, ADS, NS and NDS [71]
*S. aureus*	Influenced vs. independent	Skin	↓ FFA [75] ↓ Triglycerides [75] ↓ Long-chain ceramides [75]
Biological race	Asian vs. African vs. European		u.s.

**Table 3:** Summary of AE metabolomics studies with blood samples. Abbreviations: AE (atopic eczema participants), LS (lesional skin), NL (non-lesional skin), HE (healthy participants), P (psoriasis participants), SCSFAs (short-chain saturated fatty acids), LCSFAs (long-chain saturated fatty acids), FA (fatty acids), ADMA (asymmetric dimethyl-arginine), C (chain length). Co. (cooperation), FLG (filaggrin), FFA (free fatty acids), UT (untargeted), IV (ichthyosis vulgaris), PBMCs (peripheral blood mononuclear cell), PUFA (poly-unsaturated fatty acids), and PCA (principal component analysis). Symbols: ↑ (increase) and ↓ (decrease).

References	Matrices	Target	Sample Size	Findings
[103]	PBMCs and plasma	PUFA	20 AE 20 HE	↓ n3-PUFA in AE vs. HE ↓ Linoleic acid in AE vs. HE ↓ 12-HETE in AE vs. HE ↑ Arachidonic acid in AE PBMCs vs. HE ↓ Arachidonic acid in AE plasma vs. HE
[100]	Serum	Metabolites in Biocrates Absolute IDQ^®^ P180 kit and UT	25 AE 24 HE 13 AE 15 HE	↓ Total acylcarnitines in AE vs. HE ↓ Phosphatidylcholines in AE vs. HE No PCA separation between AE as compared to HE
[98]	Serum	Glycerophospholipids, acylcarnitines, sphingomyelins, amino acids, carbohydrates	20 AE	Only FLG (+/+) had response to drug targeting of IgE ↓ Glycerophospholipids in FLG (+/+) vs. FLG mutant ↓ Sphingomyelin in FLG (+/+) vs. FLG mutant ↓ Amino acids in FLG (+/+) vs. FLG mutant ↓ Acylcarnitines in FLG (+/+) vs. FLG mutant
[97]	Plasma	UT	58 AE 23 HE	Distinct metabolite differences for endotypes: FLG mutant and high IgE Iso-butyrate, isoleucine, tyramine, histidine, threonine, and isopropanol are associated with FLG mutation Isopropanol is associated with IgE levels
[102]	Sebum	Lipid mediators, non-esterified and total fatty acid forms	11 AE 9 HE	Sweat and sebum highly overlap in detection but concentrations of metabolites were typically higher in sebum.
[72]	Serum	FFA, eicosanoids and docosanoids	6 AE 6 HE	No change in arachidonic acid in AE vs. HE
[101]	Serum	Amine/phenol sub-metabolomes	9 AE 10 Dock8-deficient 33 HE	AE and DOCK8-deficient individuals have unique metabolomics profiles ↑ 3-hydroxyanthranilic acid in DOCK8-deficient vs. HE and AE ↑ Aspartic acid in DOCK8-deficient vs. HE ↓ Hypo-taurine in DOCK8-deficient vs. AE ↓ Glycyl-phenylalanine in DOCK8-deficient vs. AE

## References

[1] Bieber T., Akdis C., Lauener R., Traidl-Hoffmann C., Schmid-Grendelmeier P., Schäppi G., Allam J.-P., Apfelbacher C., Augustin M., Beck L. (2016). Global Allergy Forum and 3rd Davos Declaration 2015: Atopic Dermatitis/Eczema: Challenges and Opportunities toward Precision Medicine. Allergy.

[2] Asher M.I., Montefort S., Björkstén B., Lai C.K.W., Strachan D.P., Weiland S.K., Williams H., ISAAC Phase Three Study Group (2006). Worldwide Time Trends in the Prevalence of Symptoms of Asthma, Allergic Rhinoconjunctivitis, and Eczema in Childhood: ISAAC Phases One and Three Repeat Multicountry Cross-Sectional Surveys. Lancet Lond. Engl..

[3] Williams H., Stewart A., von Mutius E., Cookson W., Anderson H.R. (2008). Is Eczema Really on the Increase Worldwide?. J. Allergy Clin. Immunol..

[4] Hadi H.A., Tarmizi A.I., Khalid K.A., Gajdács M., Aslam A., Jamshed S. (2021). The Epidemiology and Global Burden of Atopic Dermatitis: A Narrative Review. Life.

[5] Nutten S. (2015). Atopic Dermatitis: Global Epidemiology and Risk Factors. Ann. Nutr. Metab..

[6] Ujiie H., Rosmarin D., Schön M.P., Ständer S., Boch K., Metz M., Maurer M., Thaci D., Schmidt E., Cole C. (2022). Unmet Medical Needs in Chronic, Non-Communicable Inflammatory Skin Diseases. Front. Med..

[7] Barbarot S., Auziere S., Gadkari A., Girolomoni G., Puig L., Simpson E.L., Margolis D.J., de Bruin-Weller M., Eckert L. (2018). Epidemiology of Atopic Dermatitis in Adults: Results from an International Survey. Allergy.

[8] Lee H.H., Patel K.R., Singam V., Rastogi S., Silverberg J.I. (2019). A Systematic Review and Meta-Analysis of the Prevalence and Phenotype of Adult-Onset Atopic Dermatitis. J. Am. Acad. Dermatol..

[9] Bantz S.K., Zhu Z., Zheng T. (2014). The Atopic March: Progression from Atopic Dermatitis to Allergic Rhinitis and Asthma. J. Clin. Cell. Immunol..

[10] Silverwood R.J., Forbes H.J., Abuabara K., Ascott A., Schmidt M., Schmidt S.A.J., Smeeth L., Langan S.M. (2018). Severe and Predominantly Active Atopic Eczema in Adulthood and Long Term Risk of Cardiovascular Disease: Population Based Cohort Study. BMJ.

[11] Ferrucci S.M., Tavecchio S., Angileri L., Surace T., Berti E., Buoli M. (2021). Factors Associated with Affective Symptoms and Quality of Life in Patients with Atopic Dermatitis. Acta Derm. Venereol..

[12] Bieber T. (2008). Atopic Dermatitis. N. Engl. J. Med..

[13] Li H., Zhang Z., Zhang H., Guo Y., Yao Z. (2021). Update on the Pathogenesis and Therapy of Atopic Dermatitis. Clinic Rev Allerg Immunol.

[14] Luschkova D., Zeiser K., Ludwig A., Traidl-Hoffmann C. (2021). Atopic Eczema Is an Environmental Disease. Allergol. Sel..

[15] Sugiura H., Ebise H., Tazawa T., Tanaka K., Sugiura Y., Uehara M., Kikuchi K., Kimura T. (2005). Large-Scale DNA Microarray Analysis of Atopic Skin Lesions Shows Overexpression of an Epidermal Differentiation Gene Cluster in the Alternative Pathway and Lack of Protective Gene Expression in the Cornified Envelope. Br. J. Dermatol..

[16] Palmer C.N.A., Irvine A.D., Terron-Kwiatkowski A., Zhao Y., Liao H., Lee S.P., Goudie D.R., Sandilands A., Campbell L.E., Smith F.J.D. (2006). Common Loss-of-Function Variants of the Epidermal Barrier Protein Filaggrin Are a Major Predisposing Factor for Atopic Dermatitis. Nat. Genet..

[17] Barnes K.C. (2010). An Update on the Genetics of Atopic Dermatitis: Scratching the Surface in 2009. J. Allergy Clin. Immunol..

[18] Agrawal R., Woodfolk J.A. (2014). Skin Barrier Defects in Atopic Dermatitis. Curr. Allergy Asthma Rep..

[19] Kobayashi T., Imanishi I. (2021). Epithelial–Immune Crosstalk with the Skin Microbiota in Homeostasis and Atopic Dermatitis—A Mini Review. Vet. Dermatol..

[20] Tabata N., Tagami H., Kligman A.M. (1998). A Twenty-Four-Hour Occlusive Exposure to 1% Sodium Lauryl Sulfate Induces a Unique Histopathologic Inflammatory Response in the Xerotic Skin of Atopic Dermatitis Patients. Acta Derm. Venereol..

[21] Flohr C., Pascoe D., Williams H.C. (2005). Atopic Dermatitis and the “Hygiene Hypothesis”: Too Clean to Be True?. Br. J. Dermatol..

[22] Ahn K. (2014). The Role of Air Pollutants in Atopic Dermatitis. J. Allergy Clin. Immunol..

[23] Wong T.-Y. (2017). Smog Induces Oxidative Stress and Microbiota Disruption. J. Food Drug Anal..

[24] Celebi Sozener Z., Ozdel Ozturk B., Cerci P., Turk M., Gorgulu Akin B., Akdis M., Altiner S., Ozbey U., Ogulur I., Mitamura Y. (2022). Epithelial Barrier Hypothesis: Effect of the External Exposome on the Microbiome and Epithelial Barriers in Allergic Disease. Allergy.

[25] Ong P.Y., Ohtake T., Brandt C., Strickland I., Boguniewicz M., Ganz T., Gallo R.L., Leung D.Y.M. (2002). Endogenous Antimicrobial Peptides and Skin Infections in Atopic Dermatitis. N. Engl. J. Med..

[26] Fyhrquist N., Muirhead G., Prast-Nielsen S., Jeanmougin M., Olah P., Skoog T., Jules-Clement G., Feld M., Barrientos-Somarribas M., Sinkko H. (2019). Microbe-Host Interplay in Atopic Dermatitis and Psoriasis. Nat. Commun..

[27] Cardona I.D., Cho S.H., Leung D.Y.M. (2006). Role of Bacterial Superantigens in Atopic Dermatitis: Implications for Future Therapeutic Strategies. Am. J. Clin. Dermatol..

[28] Hülpüsch C., Tremmel K., Hammel G., Bhattacharyya M., de Tomassi A., Nussbaumer T., Neumann A.U., Reiger M., Traidl-Hoffmann C. (2020). Skin PH-Dependent Staphylococcus Aureus Abundance as Predictor for Increasing Atopic Dermatitis Severity. Allergy.

[29] Al-Jaberi H., Marks R. (1984). Studies of the Clinically Uninvolved Skin in Patients with Dermatitis. Br. J. Dermatol..

[30] Gavrilova T. (2018). Immune Dysregulation in the Pathogenesis of Atopic Dermatitis. Dermat. Contact Atopic Occup. Drug.

[31] Yang L., Fu J., Zhou Y. (2020). Research Progress in Atopic March. Front. Immunol..

[32] Werfel T., Allam J.-P., Biedermann T., Eyerich K., Gilles S., Guttman-Yassky E., Hoetzenecker W., Knol E., Simon H.-U., Wollenberg A. (2016). Cellular and Molecular Immunologic Mechanisms in Patients with Atopic Dermatitis. J. Allergy Clin. Immunol..

[33] Guttman-Yassky E., Krueger J.G., Lebwohl M.G. (2018). Systemic Immune Mechanisms in Atopic Dermatitis and Psoriasis with Implications for Treatment. Exp. Dermatol..

[34] Czarnowicki T., He H., Krueger J.G., Guttman-Yassky E. (2019). Atopic Dermatitis Endotypes and Implications for Targeted Therapeutics. J. Allergy Clin. Immunol..

[35] Voss M., Kotrba J., Gaffal E., Katsoulis-Dimitriou K., Dudeck A. (2021). Mast Cells in the Skin: Defenders of Integrity or Offenders in Inflammation?. Int. J. Mol. Sci..

[36] Müller C., Dietz I., Tziotis D., Moritz F., Rupp J., Schmitt-Kopplin P. (2013). Molecular Cartography in Acute Chlamydia Pneumoniae Infections--a Non-Targeted Metabolomics Approach. Anal. Bioanal. Chem..

[37] Elpa D.P., Chiu H.-Y., Wu S.-P., Urban P.L. (2021). Skin Metabolomics. Trends Endocrinol. Metab..

[38] Afghani J., Huelpuesch C., Reiger M., Schmitt-Kopplin P., Traidl-Hoffmann C., Mueller C. (2021). Enhanced Access to the Health-Related Skin Metabolome by Fast, Reproducible and Non-Invasive Wet-Prep Sampling. Metabolites.

[39] Dalgard F.J., Gieler U., Tomas-Aragones L., Lien L., Poot F., Jemec G.B.E., Misery L., Szabo C., Linder D., Sampogna F. (2015). The Psychological Burden of Skin Diseases: A Cross-Sectional Multicenter Study among Dermatological out-Patients in 13 European Countries. J. Investig. Dermatol..

[40] Nestle F.O., Di Meglio P., Qin J.-Z., Nickoloff B.J. (2009). Skin Immune Sentinels in Health and Disease. Nat. Rev. Immunol..

[41] Grice E.A., Segre J.A. (2011). The Skin Microbiome. Nat. Rev. Microbiol..

[42] Byrd A.L., Belkaid Y., Segre J.A. (2018). The Human Skin Microbiome. Nat. Rev. Microbiol..

[43] Nguyen A.V., Soulika A.M. (2019). The Dynamics of the Skin’s Immune System. Int. J. Mol. Sci..

[44] Quaresma J.A.S. (2019). Organization of the Skin Immune System and Compartmentalized Immune Responses in Infectious Diseases. Clin. Microbiol. Rev..

[45] Swaney M.H., Kalan L.R. (2021). Living in Your Skin: Microbes, Molecules, and Mechanisms. Infect. Immun..

[46] Bouslimani A., da Silva R., Kosciolek T., Janssen S., Callewaert C., Amir A., Dorrestein K., Melnik A.V., Zaramela L.S., Kim J.-N. (2019). The Impact of Skin Care Products on Skin Chemistry and Microbiome Dynamics. BMC Biol..

[47] Misra N., Clavaud C., Guinot F., Bourokba N., Nouveau S., Mezzache S., Palazzi P., Appenzeller B.M.R., Tenenhaus A., Leung M.H.Y. (2021). Multi-Omics Analysis to Decipher the Molecular Link between Chronic Exposure to Pollution and Human Skin Dysfunction. Sci. Rep..

[48] Randhawa M., Southall M., Samaras S.T. (2013). Metabolomic Analysis of Sun Exposed Skin. Mol. Biosyst..

[49] Jung E.S., Park J.I., Park H., Holzapfel W., Hwang J.S., Lee C.H. (2019). Seven-Day Green Tea Supplementation Revamps Gut Microbiome and Caecum/Skin Metabolome in Mice from Stress. Sci. Rep..

[50] Kuehne A., Hildebrand J., Soehle J., Wenck H., Terstegen L., Gallinat S., Knott A., Winnefeld M., Zamboni N. (2017). An Integrative Metabolomics and Transcriptomics Study to Identify Metabolic Alterations in Aged Skin of Humans in vivo. BMC Genom..

[51] Hooton K., Han W., Li L. Comprehensive and Quantitative Profiling of the Human Sweat Submetabolome Using High-Performance Chemical Isotope Labeling LC–MS. https://pubs.acs.org/doi/pdf/10.1021/acs.analchem.6b01930.

[52] Sitter B., Johnsson M.K., Halgunset J., Bathen T.F. (2013). Metabolic Changes in Psoriatic Skin under Topical Corticosteroid Treatment. BMC Dermatol..

[53] Ono E., Murota H., Mori Y., Yoshioka Y., Nomura Y., Munetsugu T., Yokozeki H., Katayama I. (2018). Sweat Glucose and GLUT2 Expression in Atopic Dermatitis: Implication for Clinical Manifestation and Treatment. PLoS ONE.

[54] Trivedi D.K., Sinclair E., Xu Y., Sarkar D., Walton-Doyle C., Liscio C., Banks P., Milne J., Silverdale M., Kunath T. (2019). Discovery of Volatile Biomarkers of Parkinson’s Disease from Sebum. ACS Cent. Sci..

[55] Sinclair E., Trivedi D.K., Sarkar D., Walton-Doyle C., Milne J., Kunath T., Rijs A.M., de Bie R.M.A., Goodacre R., Silverdale M. (2021). Metabolomics of Sebum Reveals Lipid Dysregulation in Parkinson’s Disease. Nat. Commun..

[56] Calderón-Santiago M., Priego-Capote F., Turck N., Robin X., Jurado-Gámez B., Sanchez J.C., Luque de Castro M.D. (2015). Human Sweat Metabolomics for Lung Cancer Screening. Anal. Bioanal. Chem..

[57] Jarmusch A.K., Elijah E.O., Vargas F., Bouslimani A., da Silva R.R., Ernst M., Wang M., Del Rosario K.K., Dorrestein P.C., Tsunoda S.M. (2019). Initial Development toward Non-Invasive Drug Monitoring via Untargeted Mass Spectrometric Analysis of Human Skin. Anal. Chem..

[58] Bittremieux W., Advani R.S., Jarmusch A.K., Aguirre S., Lu A., Dorrestein P.C., Tsunoda S.M. (2021). Physicochemical Properties Determining Drug Detection in Skin. Clin. Transl. Sci..

[59] Perrier E., Demazières A., Girard N., Pross N., Osbild D., Metzger D., Guelinckx I., Klein A. (2013). Circadian Variation and Responsiveness of Hydration Biomarkers to Changes in Daily Water Intake. Eur. J. Appl. Physiol..

[60] Nam S.L., de la Mata A.P., Dias R.P., Harynuk J.J. (2020). Towards Standardization of Data Normalization Strategies to Improve Urinary Metabolomics Studies by GC×GC-TOFMS. Metabolites.

[61] Hahnefeld L., Gurke R., Thomas D., Schreiber Y., Schäfer S.M.G., Trautmann S., Snodgrass I.F., Kratz D., Geisslinger G., Ferreirós N. (2020). Implementation of Lipidomics in Clinical Routine: Can Fluoride/Citrate Blood Sampling Tubes Improve Preanalytical Stability?. Talanta.

[62] Ang J.E., Revell V., Mann A., Mäntele S., Otway D.T., Johnston J.D., Thumser A.E., Skene D.J., Raynaud F. (2012). Identification of Human Plasma Metabolites Exhibiting Time-of-Day Variation Using an Untargeted Liquid Chromatography–Mass Spectrometry Metabolomic Approach. Chronobiol. Int..

[63] Bouslimani A., Porto C., Rath C.M., Wang M., Guo Y., Gonzalez A., Berg-Lyon D., Ackermann G., Christensen G.J.M., Nakatsuji T. (2015). Molecular Cartography of the Human Skin Surface in 3D. Proc. Natl. Acad. Sci. USA.

[64] Stiemsma L.T., Reynolds L.A., Turvey S.E., Finlay B.B. (2015). The Hygiene Hypothesis: Current Perspectives and Future Therapies. Immunotargets Ther..

[65] Strachan D.P. (1989). Hay Fever, Hygiene, and Household Size. BMJ.

[66] Bloomfield S., Stanwell-Smith R., Crevel R., Pickup J. (2006). Too Clean, or Not Too Clean: The Hygiene Hypothesis and Home Hygiene. Clin. Exp. Allergy.

[67] Ege M.J., Mayer M., Normand A.-C., Genuneit J., Cookson W.O.C.M., Braun-Fahrländer C., Heederik D., Piarroux R., von Mutius E., GABRIELA Transregio 22 Study Group (2011). Exposure to Environmental Microorganisms and Childhood Asthma. N. Engl. J. Med..

[68] Bhattacharya N., Sato W.J., Kelly A., Ganguli-Indra G., Indra A.K. (2019). Epidermal Lipids: Key Mediators of Atopic Dermatitis Pathogenesis. Trends Mol. Med..

[69] van Smeden J., Bouwstra J.A. (2016). Stratum Corneum Lipids: Their Role for the Skin Barrier Function in Healthy Subjects and Atopic Dermatitis Patients. Curr. Probl. Dermatol..

[70] Ilves L., Ottas A., Kaldvee B., Abram K., Soomets U., Zilmer M., Jaks V., Kingo K. (2021). Metabolomic Analysis of Skin Biopsies from Patients with Atopic Dermatitis Reveals Hallmarks of Inflammation, Disrupted Barrier Function and Oxidative Stress. Acta Derm. Venereol..

[71] Emmert H., Baurecht H., Thielking F., Stölzl D., Rodriguez E., Harder I., Proksch E., Weidinger S. (2021). Stratum Corneum Lipidomics Analysis Reveals Altered Ceramide Profile in Atopic Dermatitis Patients across Body Sites with Correlated Changes in Skin Microbiome. Exp. Dermatol..

[72] Töröcsik D., Weise C., Gericke J., Szegedi A., Lucas R., Mihaly J., Worm M., Rühl R. (2019). Transcriptomic and Lipidomic Profiling of Eicosanoid/Docosanoid Signalling in Affected and Non-Affected Skin of Human Atopic Dermatitis Patients. Exp. Dermatol..

[73] Agrawal K., Hassoun L.A., Foolad N., Pedersen T.L., Sivamani R.K., Newman J.W. (2017). Sweat Lipid Mediator Profiling: A Noninvasive Approach for Cutaneous Research. J. Lipid Res..

[74] Blunder S., Rühl R., Moosbrugger-Martinz V., Krimmel C., Geisler A., Zhu H., Crumrine D., Elias P.M., Gruber R., Schmuth M. (2017). Alterations in Epidermal Eicosanoid Metabolism Contribute to Inflammation and Impaired Late Differentiation in FLG-Mutated Atopic Dermatitis. J. Investig. Dermatol..

[75] Li S., Villarreal M., Stewart S., Choi J., Ganguli-Indra G., Babineau D.C., Philpot C., David G., Yoshida T., Boguniewicz M. (2017). Altered Composition of Epidermal Lipids Correlates with Staphylococcus Aureus Colonization Status in Atopic Dermatitis. Br. J. Dermatol..

[76] Schäfer L., Kragballe K. (1991). Abnormalities in Epidermal Lipid Metabolism in Patients with Atopic Dermatitis. J. Investig. Dermatol..

[77] Ruzicka T., Simmet T., Peskar B.A., Ring J. (1986). Skin Levels of Arachidonic Acid-Derived Inflammatory Mediators and Histamine in Atopic Dermatitis and Psoriasis. J. Investig. Dermatol..

[78] Lee S.-J., Kim S.-E., Shin K.-O., Park K., Lee S.E. (2021). Dupilumab Therapy Improves Stratum Corneum Hydration and Skin Dysbiosis in Patients with Atopic Dermatitis. Allergy Asthma Immunol. Res..

[79] Yang J.-J., Chang T.-W., Jiang Y., Kao H.-J., Chiou B.-H., Kao M.-S., Huang C.-M. (2018). Commensal Staphylococcus Aureus Provokes Immunity to Protect against Skin Infection of Methicillin-Resistant Staphylococcus Aureus. Int. J. Mol. Sci..

[80] Callewaert C., Nakatsuji T., Knight R., Kosciolek T., Vrbanac A., Kotol P., Ardeleanu M., Hultsch T., Guttman-Yassky E., Bissonnette R. (2020). IL-4Rα Blockade by Dupilumab Decreases Staphylococcus Aureus Colonization and Increases Microbial Diversity in Atopic Dermatitis. J. Investig. Dermatol..

[81] Feingold K.R., Elias P.M. (2014). Role of Lipids in the Formation and Maintenance of the Cutaneous Permeability Barrier. Biochim. Biophys. Acta.

[82] Elias P.M. (1996). Stratum Corneum Architecture, Metabolic Activity and Interactivity with Subjacent Cell Layers. Exp. Dermatol..

[83] Holland K.T., Greenman J., Cunliffe W.J. (1979). Growth of Cutaneous Propionibacteria on Synthetic Medium; Growth Yields and Exoenzyme Production. J. Appl. Bacteriol..

[84] Brüggemann H., Henne A., Hoster F., Liesegang H., Wiezer A., Strittmatter A., Hujer S., Dürre P., Gottschalk G. (2004). The Complete Genome Sequence of Propionibacterium Acnes, a Commensal of Human Skin. Science.

[85] Nakatsuji T., Chiang H.-I., Jiang S.B., Nagarajan H., Zengler K., Gallo R.L. (2013). The Microbiome Extends to Subepidermal Compartments of Normal Skin. Nat. Commun..

[86] Jang H., Matsuda A., Jung K., Karasawa K., Matsuda K., Oida K., Ishizaka S., Ahn G., Amagai Y., Moon C. (2016). Skin PH Is the Master Switch of Kallikrein 5-Mediated Skin Barrier Destruction in a Murine Atopic Dermatitis Model. J. Investig. Dermatol..

[87] Stücker M., Struk A., Altmeyer P., Herde M., Baumgärtl H., Lübbers D.W. (2002). The Cutaneous Uptake of Atmospheric Oxygen Contributes Significantly to the Oxygen Supply of Human Dermis and Epidermis. J. Physiol..

[88] Gong J.Q., Lin L., Lin T., Hao F., Zeng F.Q., Bi Z.G., Yi D., Zhao B. (2006). Skin Colonization by Staphylococcus Aureus in Patients with Eczema and Atopic Dermatitis and Relevant Combined Topical Therapy: A Double-Blind Multicentre Randomized Controlled Trial. Br. J. Dermatol..

[89] Wang Y., Kuo S., Shu M., Yu J., Huang S., Dai A., Two A., Gallo R.L., Huang C.-M. (2014). Staphylococcus Epidermidis in the Human Skin Microbiome Mediates Fermentation to Inhibit the Growth of Propionibacterium Acnes: Implications of Probiotics in Acne Vulgaris. Appl. Microbiol. Biotechnol..

[90] Lambers H., Piessens S., Bloem A., Pronk H., Finkel P. (2006). Natural Skin Surface PH Is on Average below 5, Which Is Beneficial for Its Resident Flora. Int. J. Cosmet. Sci..

[91] Schwarz A., Bruhs A., Schwarz T. (2017). The Short-Chain Fatty Acid Sodium Butyrate Functions as a Regulator of the Skin Immune System. J. Investig. Dermatol..

[92] Krejner A., Bruhs A., Mrowietz U., Wehkamp U., Schwarz T., Schwarz A. (2018). Decreased Expression of G-Protein-Coupled Receptors GPR43 and GPR109a in Psoriatic Skin Can Be Restored by Topical Application of Sodium Butyrate. Arch. Dermatol. Res..

[93] Rutting S., Xenaki D., Malouf M., Horvat J.C., Wood L.G., Hansbro P.M., Oliver B.G. (2018). Short-Chain Fatty Acids Increase TNFα-Induced Inflammation in Primary Human Lung Mesenchymal Cells through the Activation of P38 MAPK. Am. J. Physiol. Lung Cell. Mol. Physiol..

[94] Sanford J.A., O’Neill A.M., Zouboulis C.C., Gallo R.L. (2019). Short-Chain Fatty Acids from Cutibacterium Acnes Activate Both a Canonical and Epigenetic Inflammatory Response in Human Sebocytes. J. Immunol..

[95] Morrison D.J., Preston T. (2016). Formation of Short Chain Fatty Acids by the Gut Microbiota and Their Impact on Human Metabolism. Gut Microbes.

[96] Schlatterer K., Peschel A., Kretschmer D. (2021). Short-Chain Fatty Acid and FFAR2 Activation-A New Option for Treating Infections?. Front. Cell. Infect. Microbiol..

[97] Chiu C.-Y., Lin G., Wang C.-J., Hung S.-I., Chung W.-H. (2021). Metabolomics Reveals Microbial-Derived Metabolites Associated with Immunoglobulin E Responses in Filaggrin-Related Atopic Dermatitis. Pediatr. Allergy Immunol. Off. Publ. Eur. Soc. Pediatr. Allergy Immunol..

[98] Hotze M., Baurecht H., Rodríguez E., Chapman-Rothe N., Ollert M., Fölster-Holst R., Adamski J., Illig T., Ring J., Weidinger S. (2014). Increased Efficacy of Omalizumab in Atopic Dermatitis Patients with Wild-Type Filaggrin Status and Higher Serum Levels of Phosphatidylcholines. Allergy.

[99] Matthias J., Maul J., Noster R., Meinl H., Chao Y.-Y., Gerstenberg H., Jeschke F., Gasparoni G., Welle A., Walter J. (2019). Sodium Chloride Is an Ionic Checkpoint for Human TH2 Cells and Shapes the Atopic Skin Microenvironment. Sci. Transl. Med..

[100] Ottas A., Fishman D., Okas T.-L., Püssa T., Toomik P., Märtson A., Kingo K., Soomets U. (2017). Blood Serum Metabolome of Atopic Dermatitis: Altered Energy Cycle and the Markers of Systemic Inflammation. PLoS ONE.

[101] Jacob M., Gu X., Luo X., Al-Mousa H., Arnaout R., Al-Saud B., Lopata A.L., Li L., Dasouki M., Rahman A.M.A. (2019). Metabolomics Distinguishes DOCK8 Deficiency from Atopic Dermatitis: Towards a Biomarker Discovery. Metabolites.

[102] Agrawal K., Sivamani R.K., Newman J.W. (2019). Noninvasive Profiling of Sweat-Derived Lipid Mediators for Cutaneous Research. Ski. Res. Technol..

[103] Mihály J., Gericke J., Törőcsik D., Gáspár K., Szegedi A., Rühl R. (2013). Reduced Lipoxygenase and Cyclooxygenase Mediated Signaling in PBMC of Atopic Dermatitis Patients. Prostaglandins Other Lipid Mediat..

[104] Park Y.M., Lee S.Y., Kang M.J., Kim B.S., Lee M.J., Jung S.S., Yoon J.S., Cho H.J., Lee E., Yang S.I. (2020). Imbalance of Gut Streptococcus, Clostridium, and Akkermansia Determines the Natural Course of Atopic Dermatitis in Infant. Allergy Asthma Immunol. Res..

[105] Ta L.D.H., Chan J.C.Y., Yap G.C., Purbojati R.W., Drautz-Moses D.I., Koh Y.M., Tay C.J.X., Huang C.-H., Kioh D.Y.Q., Woon J.Y. (2020). A Compromised Developmental Trajectory of the Infant Gut Microbiome and Metabolome in Atopic Eczema. Gut Microbes.

[106] Kong W.S., Tsuyama N., Inoue H., Guo Y., Mokuda S., Nobukiyo A., Nakatani N., Yamaide F., Nakano T., Kohno Y. (2021). Long-Chain Saturated Fatty Acids in Breast Milk Are Associated with the Pathogenesis of Atopic Dermatitis via Induction of Inflammatory ILC3s. Sci. Rep..

[107] Herberth G., Offenberg K., Rolle-Kampczyk U., Bauer M., Otto W., Röder S., Grützmann K., Sack U., Simon J.-C., Borte M. (2015). Endogenous Metabolites and Inflammasome Activity in Early Childhood and Links to Respiratory Diseases. J. Allergy Clin. Immunol..

[108] Lee Y., Lee E., Yon D.K., Jee H.M., Baek H.S., Lee S.W., Cho J.-Y., Han M.Y. (2021). The Potential Pathways Underlying the Association of Propyl-Paraben Exposure with Aeroallergen Sensitization and EASI Score Using Metabolomics Analysis. Sci. Rep..

[109] Huang Y., Chen G., Liu X., Shao Y., Gao P., Xin C., Cui Z., Zhao X., Xu G. (2014). Serum Metabolomics Study and Eicosanoid Analysis of Childhood Atopic Dermatitis Based on Liquid Chromatography-Mass Spectrometry. J. Proteome Res..

[110] Assfalg M., Bortoletti E., D’Onofrio M., Pigozzi R., Molinari H., Boner A.L., Peroni D.G., Piacentini G.L. (2012). An Exploratory (1) H-Nuclear Magnetic Resonance Metabolomics Study Reveals Altered Urine Spectral Profiles in Infants with Atopic Dermatitis. Br. J. Dermatol..

[111] Wang H., Cui L., Jia Y., Gao Y., Zhang G., He C. (2020). Application of Lipidomics to Reveal Differences of Facial Skin Surface Lipids between Atopic Dermatitis and Healthy Infants. J. Cosmet. Dermatol..

[112] Yang L., Sato M., Saito-Abe M., Nishizato M., Mezawa H., Yamamoto-Hanada K., Ohya Y. (2021). Serum 25-Hydroxyvitamin D Concentrations and Atopic Dermatitis in Early Childhood: Findings from the Japan Environment and Children’s Study. Nutrients.

[113] Brüggen M.-C., Stingl G. (2020). Subcutaneous White Adipose Tissue: The Deepest Layer of the Cutaneous Immune Barrier. JDDG J. Dtsch. Dermatol. Ges..

[114] Proksch E. (2018). PH in Nature, Humans and Skin. J. Dermatol..

[115] Yousaf M., Ayasse M., Ahmed A., Gwillim E.C., Janmohamed S.R., Yousaf A., Patel K.R., Thyssen J.P., Silverberg J.I. (2022). Association between Atopic Dermatitis and Hypertension: A Systematic Review and Meta-Analysis. Br. J. Dermatol..

[116] Selvarajah V., Connolly K., McEniery C., Wilkinson I. (2018). Skin Sodium and Hypertension: A Paradigm Shift?. Curr. Hypertens. Rep..

[117] Selvarajah V., Mäki-Petäjä K.M., Pedro L., Bruggraber S.F.A., Burling K., Goodhart A.K., Brown M.J., McEniery C.M., Wilkinson I.B. (2017). Novel Mechanism for Buffering Dietary Salt in Humans: Effects of Salt Loading on Skin Sodium, Vascular Endothelial Growth Factor C, and Blood Pressure. Hypertension.

[118] Hon K.L., Kung J.S.C., Ng W.G., Tsang K.Y.C., Cheng N., Leung T.F. (2021). Are Skin Equipment for Assessing Childhood Eczema Any Good?. J. Dermatol. Treat..

[119] Reilly D.M., Parslew R., Sharpe G.R., Powell S., Green M.R. (2000). Inflammatory Mediators in Normal, Sensitive and Diseased Skin Types. Acta Derm. Venereol..

[120] Behrendt H., Ring J. (1990). Histamine, Antihistamines and Atopic Eczema. Clin. Exp. Allergy J. Br. Soc. Allergy Clin. Immunol..

[121] Masini E., Giannella E., Bani-Sacchi T., Fantozzi R., Palmerani B., Mannaioni P.F. (1987). Histamine Release from Serosal Mast Cells by Intermediate Products of Arachidonic Acid Metabolism. Agents Actions.

